# Structural covariance analysis for neurodegenerative and neuroinflammatory brain disorders

**DOI:** 10.1093/brain/awaf151

**Published:** 2025-05-16

**Authors:** Neus Mongay-Ochoa, Gabriel Gonzalez-Escamilla, Vinzenz Fleischer, Deborah Pareto, Àlex Rovira, Jaume Sastre-Garriga, Sergiu Groppa

**Affiliations:** Department of Neurology, Saarland University and Saarland University Medical Center, Homburg 66421, Germany; Mutiple Sclerosis Centre of Catalonia (Cemcat), Department of Neurology, Vall d’Hebron University Hospital, Universitat Autònoma de Barcelona, Barcelona 08035, Spain; Department of Neurology, Saarland University and Saarland University Medical Center, Homburg 66421, Germany; Department of Neurology, University Medical Center of the Johannes Gutenberg University Mainz, Mainz 55131, Germany; Section of Neuroradiology, Department of Radiology, Vall d’Hebron University Hospital, Universitat Autònoma de Barcelona, Barcelona 08035, Spain; Section of Neuroradiology, Department of Radiology, Vall d’Hebron University Hospital, Universitat Autònoma de Barcelona, Barcelona 08035, Spain; Mutiple Sclerosis Centre of Catalonia (Cemcat), Department of Neurology, Vall d’Hebron University Hospital, Universitat Autònoma de Barcelona, Barcelona 08035, Spain; Department of Neurology, Saarland University and Saarland University Medical Center, Homburg 66421, Germany

**Keywords:** morphometric covariance networks, structural MRI, grey matter, neuroinflammation, neurodegeneration

## Abstract

Structural MRI can robustly assess brain tissue alterations related to neurological diseases and ageing. Traditional morphological MRI metrics, such as cortical volume and thickness, only partially relate to functional impairment and disease trajectories at the individual level. Emerging research has increasingly focused on reconstructing interregional meso- and macro-structural relationships in the brain by analysing covarying morphometric patterns. These patterns suggest that structural variations in specific brain regions tend to covary with deviations in other regions across individuals, a phenomenon termed structural covariance. This concept reflects the idea that physiological and pathological processes follow an anatomically defined spreading pattern. Advanced computational strategies, particularly those within the graph-theoretical framework, yield quantifiable properties at both the whole-brain and regional levels, which correlate more closely with the clinical state or cognitive performance than classical atrophy patterns.

This review highlights cutting-edge methods for evaluating morphometric covariance networks on an individual basis, with a focus on their utility in characterizing ageing, central nervous system inflammation and neurodegeneration. Specifically, these methods hold significant potential for quantifying structural alterations in patients with Alzheimer’s disease, Parkinson’s disease, frontotemporal dementia and multiple sclerosis. By capturing the distinctive morphometric organization of each individual’s brain, structural covariance network analyses allow the tracking and prediction of pathology progression and clinical outcomes, information that can be integrated into clinical decision-making and used as variables in clinical trials. Furthermore, by investigating distinct and cross-diagnostic patterns of structural covariance, these approaches offer insights into shared mechanistic processes critical to understanding severe neurological disorders and their therapeutic implications. Such advancements pave the way for more precise diagnostic tools and targeted therapeutic strategies.

## Teaser

Standard MRI focuses on brain structure but may not fully capture disease progression. Mongay-Ochoa *et al.* review advanced methods for mapping brain connections and identify common topographical patterns of pathology spread across different neurological disorders, including multiple sclerosis, Alzheimer's disease and Parkinson's disease.

## Introduction

In the past, the brain was considered to be organized in anatomically defined cortical regions with well differentiated functions.^1^ However, it is more accurate to conceptualize it as a hierarchically-organized system of interacting elements, or ‘networks’,^2^ which provide the physiological basis for information processing and cognition. This shift in understanding is supported by studies demonstrating that brain regions involved in distinct cognitive functions tend to develop similarly,^3^ are strongly regulated by genes,^4,5^ change over one’s lifespan^6^ and exhibit experience-related plasticity,^7^ being collectively known as the connectome.^8^ Furthermore, distinct pattern changes have been related to clinical variables or task performance measures in different neurological disorders,^9,10^ as brain areas that share anatomical and functional properties also appear to deteriorate in a more coordinated way than non-functionally related regions.^7^

Morphometric analyses based on structural MRI acquisitions are the most established tools for exploring *in vivo* brain alterations related to ageing and neurological disorders.^11,12^ However, morphometric MRI parameters alone, such as cortical volume or thickness, are not always sensitive enough to explain particular neurological symptoms or disease trajectories.^13^ Conversely, it is increasingly acknowledged that morphometric parameters from a given region have an apparent close relation to the structural characteristics of functionally related areas.^7^ This implies that variations in the structure of certain brain regions across individuals frequently co-vary with structural variations in other brain regions, a phenomenon referred to as structural covariance.^7^ The biological significance of this covariation in grey matter (GM) properties is still under debate, but it has been clearly shown that the morphometric covariance partially reflects brain connectivity itself.^14,15^ At first, morphometric covariance analysis has been employed to detect network changes in neurodegenerative disorders and to understand their role in cognitive decline, as neurodegeneration spreads in well-defined structural patterns related to proximity and neuroanatomical characteristics.^16^ However, focal or disseminated lesions (inflammatory, ischaemic, traumatic, tumoural) can also impact brain network organization,^13,17,18^ leading to global changes in large-scale brain functionality beyond structural damage.^19^ Furthermore, due to the growing recognized association of neuroinflammation and neurodegeneration across different neurological diseases,^20^ covarying morphometric patterns are being progressively explored to depict the trajectories of neuroinflammatory conditions.^21,22^

**Figure 1:**
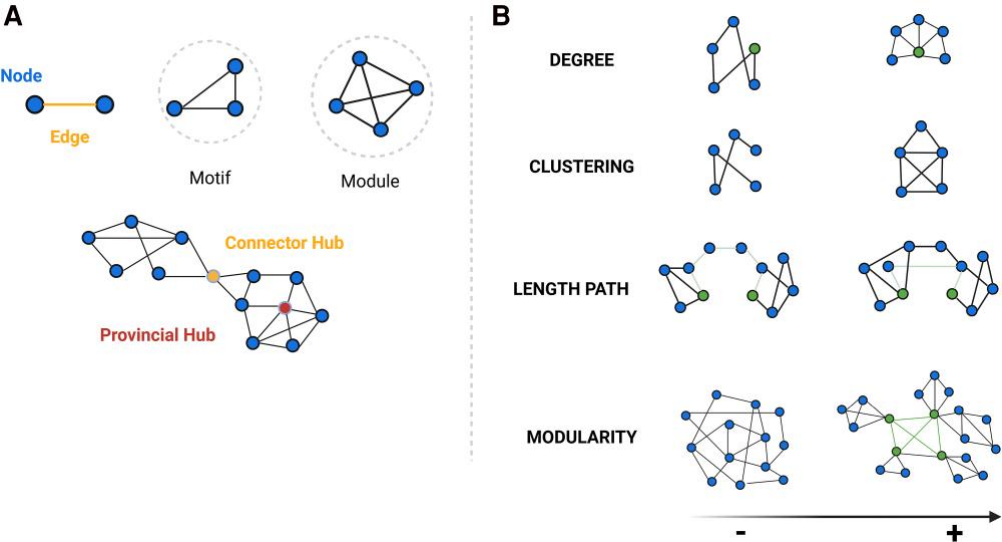
**Hierarchical organization of nodes and edges and key graph-based properties**. (**A**) Graph theory allows the categorizing of nodes according to their connections within a network (motifs, modules),^23^ organizing of nodes and edges in terms of network hierarchy (hubs),^24^ as well as (**B**) quantitative evaluation of a wide range of network properties.^19^ Some of the main properties are: node degree—the number of edges connected to a given node; cluster coefficient: the probability that any two neighbouring nodes are connected; length path—the average number of links required to traverse between all pairs of nodes in a network, showing network’s overall connectivity and efficiency in information transfer; betweenness centrality—defined as the number of shortest paths between any two nodes that passes through an any specific node and identifies those nodes that act as critical intermediaries in maintaining efficient information flow within the network; and modularity—reflecting the tendency of a network to be organized into distinct, densely connected groups of nodes or modules. This is related to scale-free network, where a few nodes have a very high node degree and betweenness centrality (hubs), while the majority of nodes display a lower node degree and the small-world network, where most nodes can be reached from every other by a small number of steps (short-path length), also exhibiting a high degree of local connections (high clustering coefficient). Studies have shown that the organizational structure of healthy human brains exhibits both non-random small-world and scale-free properties,^23^ which confer an optimal balance between local specialization and global segregation, enhancing the efficiency and resilience. Moreover, the role of hubs within brain networks is crucial for managing the majority of information traffic.^25^ Healthy brain networks also display a hierarchical modular structure,^8^ with subnetworks within larger networks. These large-scale modules correspond to recognized functional systems in the brain, including motor, somatosensory, auditory, visual and association networks.^19^ Created in BioRender. Groppa, S. (2025) https://BioRender.com/nptbd0r.

The graph-based analytical framework,^8,23^ one of the most commonly used approaches to analyse morphometric covariance networks, attempts to summarize complex global and regional covariance patterns into biologically meaningful properties, providing an abstract but quantifiable representation of their constituent elements (nodes) and connections (edges) among them. Accordingly, nodes represent neurons or brain regions, whereas edges represent synapses or axonal projections.^8,23^ (Fig. 1) In addition, graph-based properties of human networks have been linked directly to brain maturation, cognitive performance (including verbal fluency, memory and intelligence), behaviour and emotions,^23^ as well as their disruption due to ageing and certain neurodegenerative and neuroinflammatory diseases.^9^ Subsequently, these conditions could be interpreted as dysfunction of brain networks or ‘disconnection syndromes’,^26^ suggesting that the loss of neurons and their connections interferes with the structural and functional connections between brain regions, leading to clinical symptoms.

To date, most network mapping studies in humans have focused on group-level analysis, neglecting any variability among subjects, even between individuals with the same diagnosis. Conversely, several methods to characterize individual structural covariance networks across neurological disorders have already arisen. Therefore, in this review, we aimed: (i) to summarize the main methods of building individual brain networks based on co-varying morphometric parameters, (ii) to update the available evidence about its use in characterizing ageing; and (iii) to discuss its potential role in understanding the underlying pathology of neurodegenerative and neuroinflammatory processes, specifically mild cognitive impairment (MCI) and Alzheimer’s disease, Parkinson’s disease, frontotemporal dementia (FTD) and multiple sclerosis.

## Establishing methodologies

**Figure 2:**
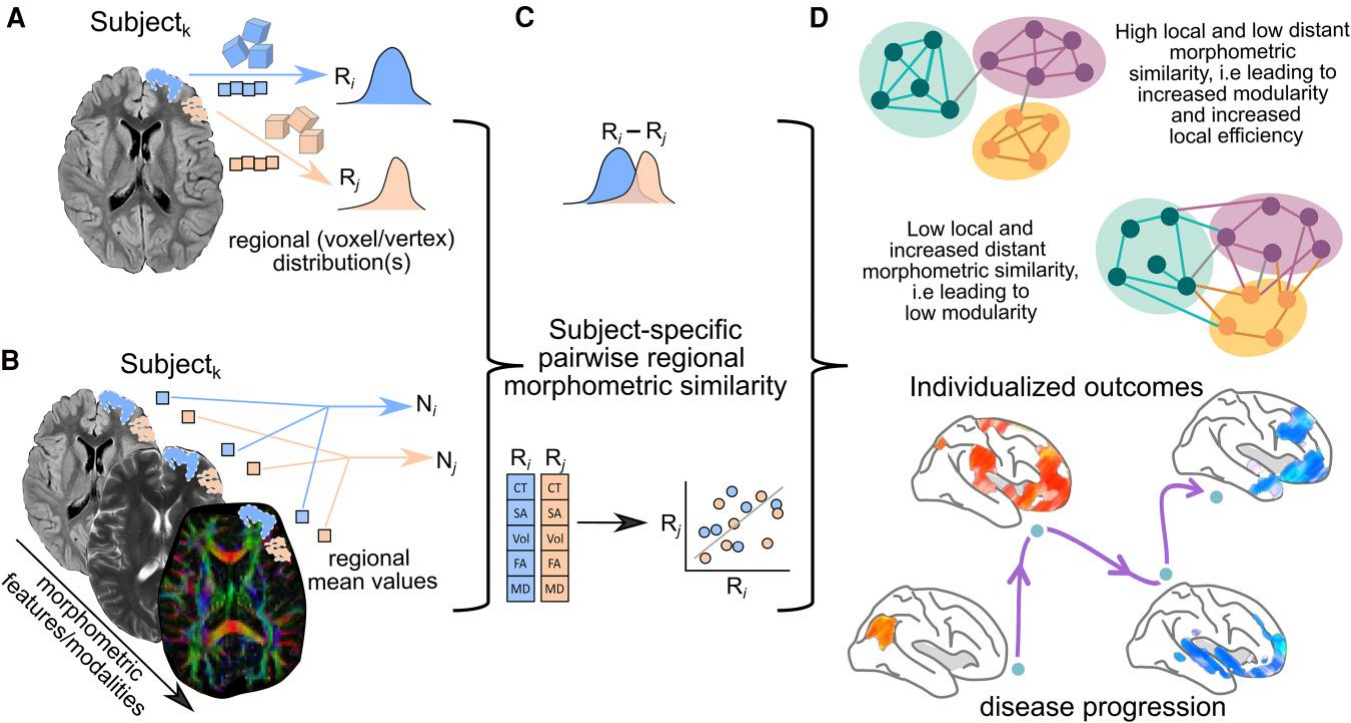
**Schematic representation of the general methodology to assess single-subject grey matter structural covariance networks**. (**A**) Individual unimodal or (**B**) multimodal MRI parameters could be used for this purpose. (**C**) Network matrices are built on pairwise similarity (known as edges or connections) between regions (known as nodes) on the extracted morphometric features (cortical thickness, volume, diffusion anisotropy, etc.). (**D**) The organization of the reconstructed network is commonly evaluated using graph theory and the resulting network parameters used to stratify phenotypes, associate with cognitive performance or assess their clinical relevance. Image partially created with BioRender.com. CT = cortical thickness; FA = fractional anisotropy; MD = mean diffusivity; SA = surface area; Vol = volume.

Overall, the common approach of the current methodologies involves constructing a structural covariance network by identifying statistically interrelated or covarying GM morphometric regions. This is achieved by transforming each individual’s set of MRI measurements into a similarity matrix of pairwise interregional correlations of morphometric feature vectors, which represents the implicit strength of these connections.^26^ This connectivity matrix can be thresholded and additionally binarized to reduce spurious or false-positive connections. Subsequently, the network is constructed based on the pairwise correlated brain regions, and the graph properties are computed for each extracted network, providing insights into the structural relationships and connectivity patterns within the brain (Fig. 2).

Differences among the available methods depend on various factors, including the cortical parcellation into nodes. This initial process typically follows a neuroanatomical scheme reflecting functional specialization. Strategies for node definition are evolving and constitute an active area of research, as the choice of the brain parcellation scheme can influence the resulting network architecture.^26^

Another aspect is morphometric measurement, as there is a range of different coupling metrics that can be estimated depending on the chosen MRI sequences.^27^ If a 3D T1-weighted image is used (unimodal MRI), the source of GM measurement is commonly cortical thickness or volume. Conversely, when employing multimodal MRI (i.e. T1-weighted, T2-weighted, diffusion-weighted data), a combination of different metrics can be calculated for each voxel.^5^ Most methods employ Pearson’s correlation to create the matrix, but alternative approaches, such as assessing the difference of absolute volumes and the Kullback–Leibler divergence (KLD) similarity,^28^ have also been explored. The latter estimates morphometric covariance between brain regions based on the difference between two probability distributions of a single morphological index. The choice of the threshold used to generate a similarity matrix from pairwise correlations is another relevant step. Common approaches include using an absolute correlation value, selecting a top percentile of correlations, applying statistical significance tests or maintaining a specific sparsity level to balance network density.^27^ Thresholds can also be adjusted to preserve important network properties and compared against random networks to ensure biological relevance.

There are also differences regarding scanner acquisition strength. A 3 T MRI provides higher signal-to-noise (SNR) and contrast-to-noise ratios between GM and white matter (WM).^29^ This results in more detailed images compared to 1.5 T scanners, potentially allowing for more precise analysis of the brain’s structural connectivity. However, studies have reported a general agreement in structural covariance networks built using both 1.5 T and 3 T scanners in terms of global network metrics.^30^ In contrast, there is poorer consistency when analysing structural connections at an individual level,^31^ although this improves when accounting for differences in network sparsity. Therefore, it is recommended to carefully consider the impact of scanner strength, especially when dealing with high-resolution data or individual connections, to ensure the reliability and comparability of structural covariance analyses across different MRI field strengths.

A wide range of graph-based network measures can be calculated from the extracted networks. Figure 1 illustrates a selection of graph metrics that are commonly used in studies of human brain networks. The primary metrics include the cluster coefficient, modularity and small-worldness; however, there is still no consensus on which metric is the most representative.^23^

Among the different methodologies for constructing structural covariance networks from morphometric GM features, the most widely used is the one described by Tijms *et al.*,^27^ which has been applied to all the neurological conditions discussed in this review. In this technique, network reconstruction is conducted by superimposing a set of precomputed 3 × 3 × 3 voxel cubes onto whole-brain GM segmentations derived from T1-weighted MRI scans. These cubes are also rotated to better accommodate the complex 3D structure of the cortex. Each cube serves as a node in the network, containing the GM volume within that specific area, while connections (edges) are established by computing correlation coefficients between pairs of cubes—the most commonly used measure of similarity.

While this cube-based method effectively accounts for brain curvature—an essential consideration given the cortex's intricate 3D structure—it does not fully capture tissue variability in shape and size across different brain regions or between the cortex and deep GM structures. Additionally, the rotating cubes may partially overlap, potentially introducing artificial increases in similarity that cannot be corrected. Nonetheless, one of the method’s strongest advantages is its independence from anatomical atlases, which enhances reliability. However, this comes at the cost of losing precise anatomical localization, thereby limiting insights into the cytoarchitectonic and myeloarchitectonic properties of brain tissue.

Other methods have emerged, such as the one described by Seidlitz *et al.*,^5^ where they employ multimodal MRI measurements to construct morphometric networks, demonstrating that any MRI metric or data from other neuroimaging techniques could be utilized for this purpose. In addition, the approach described by Gonzalez-Escamilla *et al.*^32^ employs a single morphological measure—the adjusted cortical volume difference between a pair of regions—to construct covariance networks.

Each methodology offers distinct ways to quantitatively characterize morphometric covariance networks and has been applied in various types of studies (cross-sectional versus longitudinal), encompassing different populations and research goals. Unfortunately, there is a lack of studies comparing the accuracy and reproducibility of these methods, resulting in an unknown but potentially significant heterogeneity. The main characteristics of each method are summarized in Table 1. A more comprehensive description is provided in the Supplementary material. Despite considerable methodological heterogeneity, there is an encouraging degree of convergence between studies of structural brain networks describing the fundamental architecture of interregional connections.^19^

**Table 1:** Key characteristics of the main methods for analysing individual structural covariance networks

Method	Nodes	Morphometric measures	Edges	Graph-based properties	Particularities
Tijms *et al.*^27^	Cortex segmentation into rotating cubes (6 × 6 × 6 mm^3^)	Local thickness and folding structure	Pearson’s correlation between cube pairs	Network degree, path length, clustering coefficient, betweenness centrality	Resulting networks are not normalized
Fleischer *et al.*^22^	Cortex segmentation into rotating cubes (6 × 6 × 6 mm^3^)	Local thickness and folding structure	Pearson’s correlation between cube pairs	Network degree, global efficiency, transitivity	Networks with similar degrees are used for the analysis
Seidlitz *et al.*^5^	Cortex parcellation into 308 regions (Desikan-Killiany atlas)	Ten morphometric features^a^ generating a vector	Pearson’s correlation between each possible pair of morphometric vectors	Nodal similarity, density, size and degree	Potential application to different neuroimaging modalities
Ciolac *et al.*^32^	Hippocampus parcellation into 12 subfields (Desikan–Killiany atlas)	Adjusted volume by TIV, age and scanner	Volumetric similarity between each pair of nodes	Clustering coefficient, network hub detection	Potential applications beyond the hippocampus

TIV = total intracranial volume.

^a^Fractional anisotropy, mean diffusivity, magnetization transfer, grey matter volume, surface area, cortical thickness, intrinsic Gaussian curvature, mean curvature, curved index, folding index.

## Studies of ageing and cognitive domains in healthy subjects

Individual morphometric covariance analysis has revealed consistent hub regions across subjects, including the precuneus, cingulate gyrus, dorsomedial frontal regions, inferior frontal and parietal areas, middle temporal gyrus and lateral occipital cortex.^27,28,33^ These hubs consistently show high reliability in nodal centrality measurements across repeated tests, suggesting that the hub architecture is a stable and fundamental aspect of human brain organization.

Morphometric covariance networks seem to display adaptive structural reorganization through the healthy lifespan, which indicates that they may capture biologically meaningful mechanisms involved in these developmental processes. Studies based on cortical thickness covariance have shown a linear decline in clustering coefficient and local efficiency with ageing.^34^ Similarly, other studies have reported a negative correlation with age for clustering coefficient and local efficiency in morphometric brain networks based on GM volume.^28,35^ In addition, studies with larger cohorts have revealed a non-linear relationship between morphometric covariance networks and age, showing an inverted U-shaped^36^ and a cubic age-related trajectory for path length and global efficiency.^34^

Widespread sex-effect has also been observed in morphometric covariance network properties.^37^ In particular, female subjects tend to exhibit higher clustering coefficients and lower path lengths compared to their male counterparts, suggesting greater local efficiency. Besides, structural covariance networks may serve as connectome fingerprints to identify single individuals, with reported accuracy rates exceeding 98%, even among twin subjects.^37^

More intriguingly, morphometric covarying properties of the connectome appear to correlate with individual differences in cognition. Specifically, it has been demonstrated that the node degree or hubness is connected to both verbal (vocabulary) and non-verbal (matrix reasoning) skills,^5^ assessed by the Wechsler Abbreviated Scale of Intelligence (WASI) intelligence quotient (IQ) scores. Notably, vocabulary IQ scores were found to be associated with the node degree in the left-lateralized temporal and bilateral frontal cortical areas, which are related to language functioning. Additionally, non-vocabulary IQ scores seemed to be correlated with the node degree in the bilateral primary sensory cortical areas, specialized for visual and sensorimotor processing. In a study exploring individual covarying cortical thickness in a cohort of 650 healthy subjects,^34^ a correlation was observed between nodal centrality in the left superior frontal gyrus and the superior part of the precentral sulcus with cognitive performance as assessed by the Cattell test, which measures cognitive abilities without being influenced by their cultural background, education or language skills.

## Tracking brain reorganization in mild cognitive impairment and Alzheimer’s disease

The spectrum of Alzheimer’s disease, spanning from MCI to clinical dementia, is one of the major health problems in ageing populations.^38^ Still, the pathophysiological mechanisms driving the accumulation of amyloid-β plaques and tau-related neurofibrillary tangles^38^ remain poorly understood. Emerging evidence suggests that this abnormal protein deposition triggers an activation of the innate immune system and an increase in inflammatory markers that contribute to structural damage and further propagation of misfolded proteins,^39,40^ ultimately resulting in neuronal loss and brain atrophy. While specific cortical atrophy patterns related to Alzheimer’s disease have been identified, certain clinical phenotypes with distinct cognitive profiles are not entirely explained by regional volume changes alone.^13^ Therefore, Alzheimer’s disease is increasingly conceptualized as a brain network disruption or disconnection syndrome^26^ secondary to all these mediating factors that eventually lead to cognitive decline.

Early studies in this field demonstrated a preferential involvement of hubs in brain diseases with cognitive impairment, such as MCI and Alzheimer’s disease,^41,42^ which have been studied extensively using individual morphometric covariance analysis. It has been demonstrated consistently that there is a decrease in the global-network small-world coefficient, clustering coefficient and path length in those patients, as well as a decrease in betweenness centrality in medial temporal and association parietal areas.^41,42^

Interestingly, these changes appear to be related to cognitive performance. Patients with a more severe cognitive impairment have been shown to display more random graph-based morphometric properties,^41^ as indicated by correlations between the average path length, clustering coefficient and the Mini-Mental State Examination (MMSE) scores, particularly evident in the left frontal and parietal areas. Additionally, some of these associations were modified by the age of disease onset and the cognitive domains affected.^42^ In early-onset Alzheimer’s disease patients (<65 years old), a worse memory impairment was strongly associated with low clustering coefficient and path length values, and a worse language impairment was strongly associated with a more decreased betweenness centrality as compared to late-onset Alzheimer’s disease patients (>65 years old) in the left inferior frontal operculum, left inferior parietal lobule and left precuneus, which are all integral parts of the language network. Conversely, late-onset patients showed a significant relationship between worse visuospatial impairment and decreased betweenness centrality, mainly in the posterior occipital, parietal, temporal and cingulate areas, which are known to be crucial for visuospatial processing.^42^ Of note, statistical analyses were usually adjusted for GM volume; therefore, these findings cannot be solely attributed to differences in regional atrophy measurements.^41,42^

Changes in morphometric covariance networks have also been identified as a useful marker of progressive cognitive worsening.^43^ Individuals with MCI and abnormal levels of beta-amyloid in the CSF displayed lower values for node degree, clustering coefficient, path length and the small-world property, compared to cognitively intact subjects.^43^ MCI individuals displayed more randomly organized morphometric covariance networks, suggesting a tendency toward the network dynamics observed in Alzheimer’s disease and an association with faster clinical progression.^44^ Prognostic cut-offs for several graph-based morphometric network properties have been calculated to identify MCI patients who are likely to progress to dementia over a two-year follow-up. As a result, models integrating small-world coefficients, CSF tau and hippocampal volumes showed the best performance to detect progression, with an accuracy of up to 72%.^45^

Furthermore, morphometric covariance properties, compared to other Alzheimer’s disease biomarkers such as total GM volume, CSF total tau and MMSE scores, appeared to better predict hippocampal atrophy rates.^46^ Interestingly, the above-mentioned traditional biomarkers showed no association with individual rates of hippocampal atrophy, suggesting that network properties may better capture changes during very early preclinical stages. Notably, in brain regions where amyloid tends initially to aggregate, such as the anterior cingulate and precuneus, disrupted network measures (characterized by low clustering coefficient and high path length values) not only predicted faster atrophy within those regions, but also in distant regions connected to the initial sites of amyloid deposition.^46^ Therefore, morphometric covariance network changes may predict disease progression in the early stages, even before brain atrophy becomes evident.

Moreover, it has been observed that disruption in structural covariance networks accelerates with higher tau retention,^44^ as measured by PET scan, in the preclinical stages and MCI. Besides, a negative correlation has been reported between tau-retention, clustering coefficient and node degree in the posterior cingulate, inferior parietal lobule and precuneus in Alzheimer’s disease patients compared to cognitively preserved, age-matched healthy controls.^47^ Additionally, tau-PET retention has been reported to be related to greater GM network disruption in individuals across the Alzheimer’s disease continuum,^48^ more evident with increasing disease severity and tau load.

## Quantifying structural alterations in Parkinson’s disease

Parkinson’s disease stands as the second most prevalent neurodegenerative disorder,^49^ affecting 2%–3% of individuals ≥65 years old. The neuropathologic hallmark is neuronal loss in the substantia nigra, resulting in a striatal dopamine deficiency, and the presence of intracellular inclusions containing aggregates of α-synuclein, which eventually extends to the entire cerebral cortex.^49^ Initially considered solely a neurodegenerative disorder, Parkinson’s disease is now recognized as a multisystem brain disorder marked by significant neuroinflammation and immune dysfunction,^50^ both contributing to α-synuclein propagation and neuronal death,^16^ as well as being implicated in the development of several non-motor symptoms. In this complex scenario, integrated analysis of whole brain morphometric covariance networks has revealed insightful findings, enriching the understanding of the disease's evolution.

Compared to healthy subjects, Parkinson’s disease patients displayed significant changes in the graph-based morphometric networks in the early stages of the disease.^51^ These abnormalities included increased measures of network segregation, as evidenced by increased clustering coefficient and local efficiency, reflecting a loss of global efficiency. Additionally, Parkinson’s disease patients showed changes in nodal centralities, particularly in the putamen and temporal-occipital regions.^51,52^ Individual network analysis revealed an inverse correlation between nodal centralities in the right postcentral gyrus and motor disability, assessed using the Unified Parkinson’s disease Rating Scale (UPDRS) III scores, as well as disease severity, estimated by the Hoehn and Yahr stage. Parkinson’s disease patients also showed lower nodal centralities in the superior occipital gyrus and inferior temporal gyrus, which comprise the visuoperceptive pathway responsible for representing complex object features and facial perception.^51^ Altogether, these findings suggest that, initially, Parkinson’s disease patients seem to be able to uphold overall information transfer, but as the disease progresses, the brain networks gradually lose the ability to maintain global integration, ending up in a disconnection syndrome as in Alzheimer’s disease.

Furthermore, morphometric covariance networks exhibited promising potential for accurately distinguishing Parkinson’s disease patients from healthy subjects (73.1% and 72.7% accuracy, respectively). Additionally, they showed good efficacy in classifying tremor-dominant and akinetic–rigid motor subtypes with a significant accuracy of 67%.^51^

Although age significantly influences the clinical features of Parkinson’s disease patients, its role remains controversial in terms of GM covariance networks. One study found that the individual network connectivity patterns of these patients change with age,^53^ while another did not observe significant changes.^54^

Despite these findings, most studies use only a limited set of morphometric parameters and are conducted in small cohorts. Therefore, further research is needed to validate and expand upon the current observations.

## Brain alterations in patients with frontotemporal dementia

The behavioural variant of FTD is the second most common early-onset dementia,^55^ after Alzheimer’s disease. The primary clinical manifestations of bvFTD involve alterations in the regulation of personal and social cognition, reward processing and language, accompanied by prominent executive dysfunction and, in some cases, memory impairment.^56^ Histopathological features are heterogeneous, including the presence of the tau-protein, the transactive response DNA-binding protein 43 or the fused in sarcoma protein in the brain.^56^ Concurrently, there is chronic neuroinflammation and prolonged activation of microglia and astrocytes,^57^ leading to an alteration of neuronal homeostasis and uncontrolled production of pro-inflammatory factors, perpetuating ongoing neurodegenerative processes.^58^ Despite sharing some atrophy patterns with Alzheimer’s disease, bvFTD is characterized by predominant prefrontal and/or anterior temporal cortex atrophy.^55^ Nevertheless, clinical symptoms cannot be solely attributed to the volume loss in these areas.^13^

Individual morphometric covariance networks have also revealed interesting disease-related characteristics. Compared to healthy subjects, bvFTD demonstrated a lower degree of connectivity density, clustering coefficient, path length, betweenness centrality and small-worldness^55^ values using the Tijms and coworkers method.^27^ Other studies constructing networks based on cortical thickness confirmed these findings.^59^ In comparison to Alzheimer’s disease patients, bvFTD exhibited a lower clustering coefficient in the left angular gyrus and less GM volume in the left thalamus.^55^ Additionally, cognitive impairment, as measured by the MMSE score, showed the strongest correlation with morphometric network changes in the left angular gyrus, right precuneus and insula.^55,59^ These affected heteromodal association areas are known to play a crucial role in executive control, working memory and emotion processing, which are usually disrupted in bvFTD.^56^

As illustrated, bvFTD shows anatomically distinct morphometric network abnormalities, which may be linked to the underlying pathology and correlate with the cognitive performance of these patients.

## Structural network alterations in multiple sclerosis

Multiple sclerosis is the most prevalent neuroinflammatory disease of the central nervous system.^60^ It is a chronic inflammatory demyelinating disorder that results in focal and disseminated lesions in both GM and WM.^60^ Additionally, growing evidence suggests that, even from disease onset,^61^ diffuse neurodegenerative processes throughout the brain and spinal cord coexist within a context of acute inflammation, contributing to irreversible and long-term disability accumulation, leading to both cognitive and physical impairment.^62^ This impairment arises from disrupted neuronal conduction due to WM lesions in key white matter tracts,^63^ which compromise the functional integrity of widely distributed brain regions,^64^ alongside the progressive accumulation of widespread grey matter abnormalities, causing axonal loss even in areas that appear normal on conventional MRI. Therefore, multiple sclerosis can also be conceptualized as a disconnection syndrome.^26,63^

Although traditional MRI metrics, such as WM lesion volumes and global and regional atrophy, are associated with cognitive decline, they only account for part of the variability in cognitive performance.^62^ This limitation likely arises because, among other factors, these measurements do not consider the inherent interregional structural relationship of the brain.^62^ In this context, morphometric covariance networks have been also used to explore the cognitive dysfunction in multiple sclerosis at the individual level.

Multiple sclerosis patients with cognitive impairment were reported to exhibit lower values of clustering coefficient and path length, indicating a more random network topology.^62^ These findings were associated with poorer global cognitive functioning, as well as with deficits in executive function, verbal memory, information processing speed, working memory and attention.

At a regional level, network abnormalities were most prominently linked to impaired global cognition in the right frontal superior gyrus, right amygdala, left middle cingulate and left paracentral lobule—areas implicated in visual, categorical and semantic recognition.^62^ Therefore, the presence of a more random network topology in multiple sclerosis patients appears to be related to cognitive impairment, explaining the variance beyond conventional MRI and volumetric measures.

Interestingly, patients classified as having clinically isolated syndrome (CIS) already displayed distinct changes in individual structural networks. In particular, CIS patients demonstrated a higher small-world coefficient compared to healthy controls,^65^ indicating a more regular network. This suggests a tendency towards possessing dense local connections (high clustering coefficient) between nodes at the expense of long-distance connections (low path length), which may compromise the efficient balance between short and long-range information transfer.^65^

The hippocampus, crucial for cognitive functions, operates within interconnected networks. In multiple sclerosis, focal damage disrupts these networks leading to cognitive impairment.^32^ Analysing a large cohort of multiple sclerosis patients and healthy subjects, individual hippocampal networks based on volumetric variations revealed significant differences in hippocampal subfield integrity between the two groups and also among male and female patients.^32^ Specifically, multiple sclerosis patients exhibited a more clustered hippocampal network topology compared to healthy controls and this difference was more pronounced in female patients. Over time, multiple sclerosis patients developed an even more clustered network architecture along with widespread regional subfield atrophy, notably also more extensive in female patients. Additionally, the described hippocampal network and anatomical organization correlated with cognitive performance, assessed using the Paced Auditory Serial Addition Test and the Multiple Sclerosis Inventory of Cognition test. Intriguingly, these correlations were also stronger in females than in male multiple sclerosis patients.^32^

Network reorganization is a dynamic process that can be captured by GM structural network metrics. Cognitive rehabilitation has been shown to improve local efficiency in multiple sclerosis patients with advanced disease,^24^ evidenced by a significant increase in the clustering coefficient in frontal and temporal areas. This is accompanied by a significant decrease in path length in the right parietal lobe and global betweenness centrality.^24^ These structural connectivity changes following cognitive training support the positive effects of rehabilitation across all stages of the disease.

## Beyond T1-weighted MRI for connectivity network mapping

The main scope of this review was to assess individual structural covariance networks using graph theory applied to T1-weighted imaging.^23^ However, different neuroimaging modalities have also been employed to characterize network organization in terms of structural and functional connectivity within a graph-theoretical framework.^19^

Structural connectivity refers to the physical (i.e. anatomical) interconnections between brain regions, primarily represented by WM tracts. Diffusion-weighted MRI (DWI) maps these axonal pathways by capturing microstructural tissue properties and fibre orientation.^25^ In DWI-based networks, nodes represent regions from an atlas, and edges correspond to streamlines between these regions.^66^ Noteworthy, similar to T1-weighted imaging, several methodologies have been proposed to model covariance networks based on microstructural properties derived from DWI.^66-69^ However, most studies construct group-level networks rather than individual ones. DWI-derived networks consistently reveal highly clustered cortical organization, with pathways primarily linking spatially related regions through hub nodes, facilitating efficient global communication.^23^ Conversely, DWI cannot determine connection directionality, resulting in undirected graphs^66^ and struggles to accurately resolve fibre crossings, mergers and divergences, as well as small U-shaped fibres.^69^ Consequently, this can lead to incomplete connectivity profiles in certain brain regions or restrict analyses to larger WM tracts. Higher-resolution scans improve white matter representation but reduce the signal-to-noise ratio (SNR), affecting fibre tracking reliability.^66,70^ More importantly, DWI only infers anatomical connections without confirming functional activity,^71^ requiring functional neuroimaging or electrophysiology for a complete connectome analysis.

Functional connectivity is inferred from statistical dependencies between neuronal activity patterns in distinct brain regions and can be assessed using both spontaneous (resting-state) and task-evoked fluctuations measured by functional MRI (fMRI).^72^ Models derived from fMRI reveal large-scale functional networks that exhibit fundamental graph-theoretical properties,^73^ such as small-world organization and scale-free degree distribution.^23^ In functional networks, nodes represent brain regions, typically defined using an atlas, while edges correspond to the correlations in time-series signals between regions, commonly measured through blood oxygen level-dependent (BOLD) signals.^73^ Constructing fMRI-based networks requires careful methodological choices, including fMRI pre-processing pipelines, parcellation schemes, preprocessing steps and frequency band selection, all of which impact network topology.^74^ Moreover, the interpretability of these networks remains constrained by the still poorly understood physiological underpinnings of the BOLD signal.

The conditions under which fMRI data are acquired are also relevant. Recent research using a two-stage analysis approach that integrates inter-subject and intra-subject correlation analyses has revealed distinct connectivity dynamics across brain regions during natural auditory stimulation.^75^ While the primary auditory cortex exhibited stable connectivity patterns, higher-order networks, such as the stress modulation and auditory language networks, showed greater inter-individual variability.^75^ Moreover, the visuomotor control network was influenced by eyes-open versus eyes-closed conditions, highlighting the interaction between auditory and visual processing.^75^ Additionally, physiological factors, such as cerebral blood flow, metabolic rate of oxygen and blood volume, modulate the BOLD signal,^76^ ultimately affecting functional connectivity estimates. External influences, including drug use, can also induce changes in the BOLD signal; for instance, ketamine has been shown to reduce connectivity in key networks like the salience, auditory and default mode networks.^77^

In the field of molecular imaging, PET has been employed to assess functional—or metabolic^78^—connectivity using graph theory, both at the group and individual levels.^79,80^ This approach has been applied to tracers such as ^18^F-FDG (glucose metabolism), ^18^F-FDOPA (dopamine synthesis) and ^11^C-SB217045 (serotonin 5HT4 receptor density). The method involves identifying molecularly interconnected brain regions by analysing correlations in tracer uptake, assuming that stronger correlations indicate stronger shared molecular properties. PET connectivity has been used at both regional and voxel levels to study neurotransmitter systems and enzymatic activity, offering a deeper understanding of the brain's structural-functional architecture and biological alterations in brain diseases.^81-83^ While fMRI offers higher spatial and temporal resolution and eliminates radiation exposure,^76^ PET provides essential information for receptor imaging and clinical applications, providing unique insights into molecular and metabolic brain function. Of note, the reconstruction of covariance networks from PET data follows the same methodology as that used for T1-weighted imaging morphometric and DWI-derived microstructural features.

Overall, DWI tractography and fMRI are the most commonly used techniques for constructing brain networks. Conversely, structural MRI has gained increasing attention due to its high SNR, a relative insensitivity to artefacts, an increased spatial sensitivity and accessibility in clinical settings.^33^ Early studies primarily focused on group-level structural covariance networks,^84,85^ which assess morphometric correlations across participants, thereby reducing the influence of outliers and anatomical variability. While this approach provides insights into shared network properties, it inherently assumes a homogeneous covariance structure within each group, potentially overlooking subject-specific variations. This limitation has driven a methodological shift toward individual-level network analyses,^33,34,44^ which enable a more granular characterization of structural connectivity patterns.

The next advancement in network-based analyses is the introduction of multimodal image covariance approaches, which offer a comprehensive framework for studying brain connectivity by integrating structural and functional neuroimaging data.^79^ Graph theory serves as a unifying framework, providing common network measures that facilitate comparisons between structural and functional connectivity.^86^ Research has shown that structural connectivity strength is moderately predictive of functional connectivity patterns,^86^ as white matter pathways tend to connect neuronal populations with synchronized activation patterns. Structurally connected cortical regions exhibit stronger and more consistent functional connectivity than unconnected regions. In addition, studies reveal moderate coupling of age-related changes in structural and functional connectivity across the lifespan,^87^ as well as altered structural and functional connectivity patterns in neuropsychiatric disorders,^88-90^ highlighting their relevance in both normal ageing and disease. Beyond structure-function relationships, brain metabolic covariances observed in PET imaging align with neural networks identified through resting-state fMRI analyses.^91^ Furthermore, nearly 50% of PET covariance connections are associated with underlying white matter tracts assessed by DWI,^92^ and 80% of intralobar PET covariance connections appear to have a structural substrate.^92^

This expanding field holds promise for novel insights into brain diseases that cannot be fully understood through single-modality imaging alone. Integrating multi-modal neuroimaging could provide a more comprehensive understanding of how structural disruptions in brain networks contribute to functional deficits,^79^ with significant implications for neurological disorders.

## Neuroinflammation, neurodegeneration and connectivity loss

Neurodegenerative diseases are characterized by a complex interplay between GM atrophy, connectivity loss, disease progression and neuroinflammation,^20^ which together drive cognitive and functional decline. Atrophy, particularly in subcortical and associative cortical regions, reflects irreversible neuronal loss and is a hallmark of disease progression in disorders such as multiple sclerosis, Alzheimer’s disease, Parkinson’s disease and FTD. However, connectivity loss often precedes significant atrophy, disrupting large-scale brain networks and accelerating disease progression.^93^

Network-based analyses have demonstrated that neurodegeneration spreads along intrinsic connectivity pathways,^94^ leading to progressive network disintegration. For instance, in multiple sclerosis, WM lesions disrupt key connections between subcortical and cortical regions, particularly in the putamen and occipital-parietal networks,^95^ impairing processing speed. Similarly, in Parkinson’s disease, cortical thinning follows connectivity patterns, with disease progression being more pronounced in regions highly connected to early atrophy sites.^94^ In Alzheimer’s disease, functional connectivity loss within the default mode network correlates more strongly with cognitive decline than atrophy alone, highlighting its predictive value. In FTD, subtype-specific atrophy patterns drive distinct clinical symptoms^96^: bvFTD affects the frontal and anterior temporal lobes, disrupting executive function and personality; semantic variant of primary progressive aphasia (PPA) impairs semantic memory via anterior temporal lobe atrophy; and non-fluent variant of PPA affects frontal-insular circuits, leading to speech deficits.^97^ Structural and functional connectivity loss in WM tracts such as the uncinate and superior longitudinal fasciculi further exacerbates language and cognitive dysfunction.^96,97^

Neuroinflammation plays a dual role, both contributing to disease progression and triggering compensatory mechanisms. While inflammatory processes in multiple sclerosis accelerate neuronal damage and demyelination,^95^ they may also transiently increase functional connectivity as a compensatory response in early disease stages.^26,62^ Similarly, in Alzheimer’s disease, neuroinflammation driven by amyloid and tau pathology influences both structural atrophy and synaptic dysfunction, further exacerbating network disruption.^98^ Taken together, these findings suggest that atrophy and connectivity loss are interconnected processes shaped by disease-specific mechanisms, with neuroinflammation acting as a key modulator of disease progression.

## Final remarks and future directions

Despite the distinct pathophysiological mechanisms of neurodegeneration and neuroinflammation, these processes share overlapping molecular pathways,^20^ including oxidative stress, mitochondrial dysfunction, excitotoxicity and blood–brain barrier disruption.^99,100^ Moreover, while the specific causes of neuronal damage—ranging from misfolded protein accumulation in neurodegenerative diseases to autoimmune-mediated attacks in neuroinflammatory conditions—differ substantially, network analysis consistently captures common alterations in large-scale brain structure,^5,15^ interregional interactions and connectivity, which robustly correlate with cognitive and physical decline.^7,9^ Therefore, the study of neuroinflammatory and neurodegenerative disorders from a network perspective provides a unifying framework to identify common structural alterations, reflected in the topographical spread of pathology across different neurological diseases.^16^

In many brain disorders, atrophy patterns and lesion load alone do not fully explain clinical manifestations,^13^ as cognitive impairment and functional decline depend not only on localized neuronal loss, but also on how different brain areas interact and reorganise functionally to compensate for damage.^7^ Thus, progressive neuronal loss—typically more pronounced in specific brain regions whose vulnerability is determined by disease pathology—leads to observable volume reductions and morphological changes. These changes, captured by T1-weighted imaging, contribute to disruptions in brain network architecture. This highlights the need for substantial efforts to better understand how network dynamics evolve in response to disease and how the brain compensates to maintain global function despite progressive injury.

**Figure 3:**
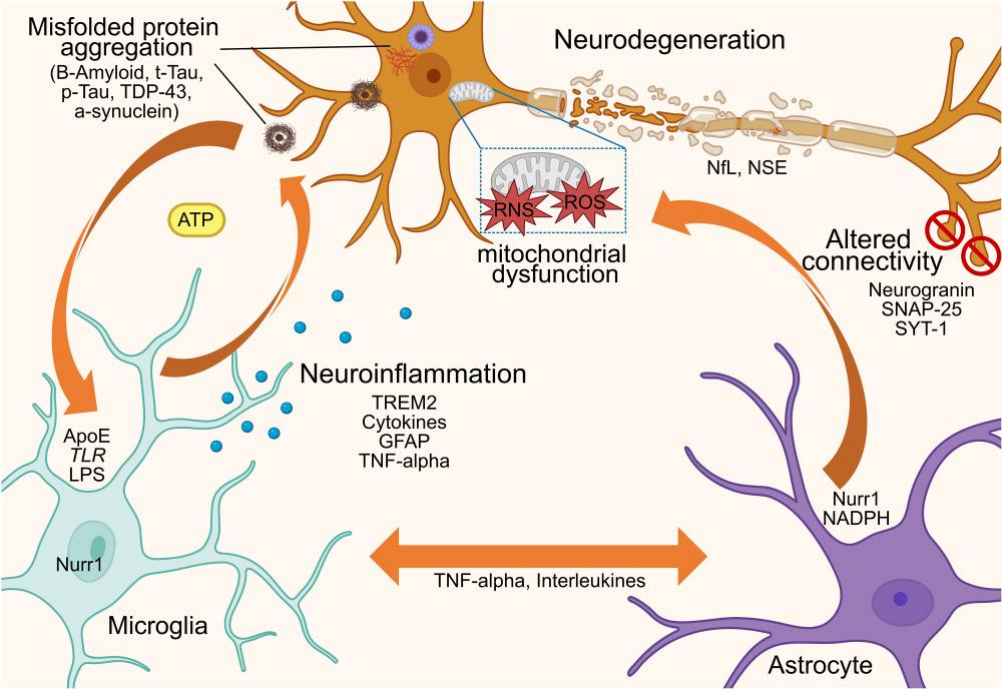
**Conceptual diagram illustrating that, in the context of brain diseases, an abnormal interplay occurs between neurons, astrocytes and microglia, driven by a self-amplifying detrimental feedback loop through the release of cytokines and neurotransmitters**. This interaction underpins the distinct yet interdependent mechanisms of neuroinflammation and neurodegeneration, which trigger and sustain misfolded protein accumulation and/or autoimmune-mediated attacks, ultimately leading to neuronal damage, network dysfunction and connectivity loss. NSE = neuron-specific enolase; RNS = reactive nitrogen species; ROS = reactive oxygen species; p-Tau = phorsphorylated tau; T-tau = total tau.

Neurodegenerative disorders are now widely known to exhibit chronic neuroinflammation, which accelerates protein aggregation and neuronal loss,^20^ thereby exacerbating disease progression. Conversely, in neuroinflammatory conditions such as multiple sclerosis,^10,17^ growing evidence suggests that neurodegenerative processes begin as early as the first demyelinating attack. These interconnected processes, including axonal damage, synaptic dysfunction and microglia activation (Fig. 3), lead to progressive motor and cognitive impairment.^62^ Despite their differences, both disease types disrupt central nervous system homeostasis, contributing to a self-perpetuating cycle of neurodegeneration marked by abnormal protein deposition, inflammatory responses and progressive neuronal death.^20^

In this context, we focused on the main available methods to construct individual morphometric covariance networks based on structural MRI due to its availability in clinical settings, high signal-to-noise ratio and reduced susceptibility to artefacts.^33^ Mapping *in vivo* GM morphometric networks has proven to offer a quantitative description of brain structural changes across the human lifespan,^34^ as well as to unravel underlying reorganization following neuronal loss due to neurodegenerative or neuroinflammatory disorders.^9^ Despite the relevant differences among the methodologies, findings from each approach have provided insightful observations on structural network reorganization, converging toward a consistent direction and providing complementary support for this morphometric covariance network-based framework. However, standardized methods are needed to facilitate the reproducibility of results across studies and validate potential clinical applications of network fingerprints for therapeutic interventional trials.

**Figure 4:**
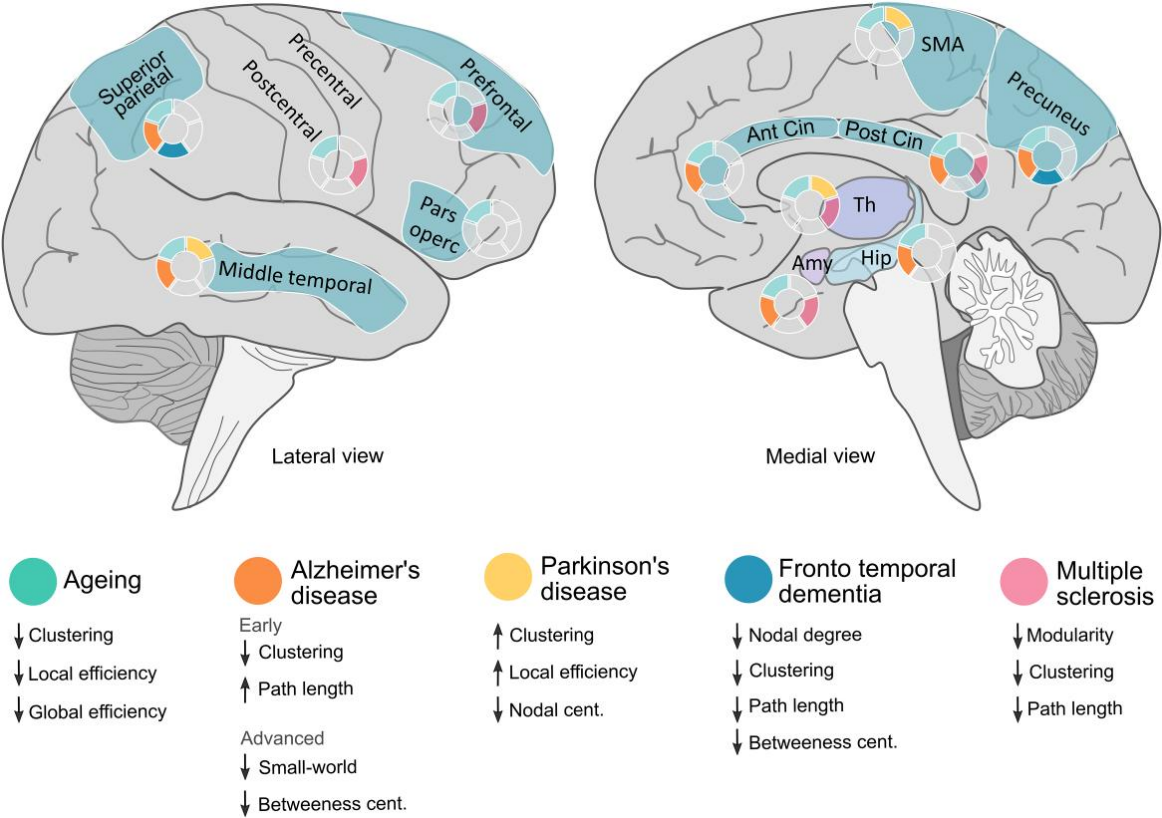
**Overview of the main morphometric covariance network changes in specific brain areas across different diseases and physiological ageing**. Lateralized changes have not been considered in this figure (see main text for further details). Amy = amygdala; Ant = anterior; Cent = centrality; Cin = cingulate; Hip = hippocampus; SMA = supplementary motor area; Th = thalamus.

Interestingly, morphometric covariance network changes observed in both neuroinflammatory and neurodegenerative disorders seem to share many similarities (Fig. 4), which may reflect the shared molecular pathways between these processes (Fig. 3). Broadly, structural network analysis offers valuable insights into disease progression, demonstrating that—independent of etiology—structural connectivity alterations follow characteristic patterns, which consist of hub overload and failure and a disruption of the hierarchical modular organization.^19^ This disruption is evidenced by loss of the characteristic non-random small-world and scale-free properties observed in healthy human brain networks.^23^ Eventually, this results in an imbalance between local processing and global efficiency, a hub overload, and ultimately, a network collapse, resulting in inefficient information flow.

Age-related network changes are understood as a physiological and dynamic process throughout the lifespan.^9^ This phenomenon is represented by changes in morphometric structural covariance networks,^27^ based upon the fact that GM organization undergoes significant structural changes with age, including synaptic proliferation, pruning and eventual atrophy. In late adulthood, the graph-based properties of structural covariance networks reveal a shift in the organization of cognitive networks from a more distributed to a more localized topological arrangement.^30,33^ This shift is attributed to the nonlinear reduction in structural associations, aligning with the disconnection syndrome hypothesis.^2^ It suggests that long-range connections may be more vulnerable to ageing effects than short-range connections,^101^ which seem to reflect individual changes in cognitive and executive functions in elderly subjects.^34^

Despite the fact that structural covariance network analysis at the individual level may capture dynamic network reorganization due to ageing, disease worsening and cognitive impairment, these methodologies have inherent limitations that should be considered when interpreting results. First, they rely heavily on accurate GM segmentation, making it susceptible to segmentation errors, particularly at tissue boundaries. While cube-based approaches^22,27^ reduce biases from traditional anatomical parcellation, they may still introduce arbitrary boundaries that do not necessarily align with functional or anatomical regions. Furthermore, the definition of network nodes, selection of morphometric features and thresholding strategies for binarizing similarity matrices can all lead to different network topologies,^26^ further contributing to variability in results across cohorts or studies. In addition, the methodology, similar to functional connectivity, assumes that morphometric similarity reflects shared properties between regions. Unlike DWI, which directly maps WM fibre tracts, structural covariance networks infer that when two regions exhibit similar structural properties, they likely share molecular or functional characteristics, which enables the quantification of pathology spread across different regions or their parallel involvement in the eloquent processing of the same brain functions.^64^ Noteworthy, while evidence suggests that structural similarity aligns with characteristic cytoarchitectonic and morphometric features, as well as aspects of axonal connectivity,^5^ the precise biological mechanisms underlying morphometric similarity remain incompletely understood.

Biological traits, including individual variability in brain anatomy or disease presentation, can also contribute to heterogeneity in network estimations. In fact, recent research has shown that the human brain exhibits an anterior-posterior gradient of microstructural asymmetry,^102^ with superficial layers displaying anterior-posterior asymmetry, while deeper layers follow an inferior-superior pattern. However, despite the regional nature of structural covariance networks, which allows them to capture asymmetry by inferring the presence or absence of corresponding regions across hemispheres, most studies do not explicitly analyse left-right differences. To address this limitation, asymmetry indices can be incorporated into network analyses to investigate how individual differences in hemispheric specialization influence network-level structural relationships.^103,104^ These key methodological issues emphasize the need for standardization, rigorous validation and complementary analytical approaches to enhance the robustness and interpretability of SCN findings.

In conclusion, the structural covariance network emerges as a valuable complementary tool to better characterize various aspects of the healthy brain. Moreover, it represents a promising approach for elucidating the neural substrates underlying diverse neurological conditions. Furthermore, it offers an avenue for jointly evaluating brain reorganization resulting from the interplay of both neurodegenerative and neuroinflammatory mechanisms. Although there is currently no standardized method for constructing morphometric covariance networks, findings from available methodologies help expand our understanding of how different pathological neurological diseases affect global brain functioning. As we gain deeper insights into the network's dynamics during ageing and brain disorders using network science, graph-based measures would presumably serve as biomarkers for tracking disease evolution and provide a window into adaptive and maladaptive reorganization processes, which could be leveraged to develop targeted interventions aimed at preserving functional integrity despite ongoing neurodegeneration.

## Supplementary Material

awaf151_Supplementary_Data
