# Supplementary material for: Structural covariance analysis for neurodegenerative and neuroinflammatory brain disorders
Source: Brain. 2025 May 16;148(9):3072–84. doi: 10.1093/brain/awaf151 (PMC12404781; doi:10.1093/brain/awaf151)
Supplement: awaf151_Supplementary_Data [file awaf151_supplementary_data.pdf]

Supplementary material: Table 1 Comprehensive description of the main methods for analysing structural covariance networks

Method	MRI	Nodes	Morphometric measures	Edges	Matrices	Graph-based properties	Target population	Particularities
Tijms et al.[25]	T1WI in a 1.5 T scan (baseline – 6 months follow-up)	Cortex segmentation into rotating cubes (6 x 6 x 6 mm^3)	Local thickness and folding structure	Pearson's correlation between cube pairs	Binarized with a threshold determined for each subject based on the significance of the correlations	Network degree, path length, clustering coefficient, betweenness centrality	Healthy controls, people with Alzheimer's disease, frontotemporal dementia and multiple sclerosis	Resulting networks are not normalized, so they varied in size across subjects
Fleischer et al.[22]	3D T1WI and 3D FLAIR in a 3T scan (baseline – 1 year follow-up)	Cortex segmentation into rotating cubes (6 x 6 x 6 mm^3)	Local thickness and folding structure	Pearson's correlation between cube pairs	Binarized with a threshold calculated as the proportion of connections that allowed the network to be fully connected	Network degree, global efficiency, transitivity	Healthy controls and people with relapsing-remitting multiple sclerosis	Networks with similar degree are used for the analysis, so groups differences do not depend on differing number of nodes or edges among subjects
Seidlitz et al.[5]	Multiparametric mapping MRI sequences and DWI in a 3T scan (baseline)	Cortex parcellation into 308 regions (Desikan-Killiany atlas)	10 morphometric features[a] generating a vector	Pearson's correlation between each possible pair of morphometric vectors	Binarized with a threshold, so as to the strongest edge was set equal to 1, and all others to 0	Nodal similarity, density, size and degree	Healthy controls and a macaque monkey cohort	Potential application to different neuroimaging modalities (multimodal MRI, PET, etc)
Ciolac et al.[30]	3D T1WI and 3D FLAIR in a 3T scan (baseline – 2 years follow-up)	Hippocampus parcellation into 12 subfields (Desikan-Killiany atlas)	Adjusted volume by TIV, age, and scanner	Volumetric similarity between each pair of nodes	Given 12 hippocampal subfield in each hemisphere, a 24x24 matrix was built for each subject	Clustering coefficient, network hub detection	Healthy controls and people with multiple sclerosis	Potential applications beyond the hippocampus

DWI, diffusion-weighted imaging; FLAIR, fluid-attenuated inversion recovery; TIV, total intracranial volume; T1WI, T1-weighted imaging; mm^3, cubic millimetres; PET, positron emission.

[a] Fractional anisotropy, mean diffusivity, magnetization transfer, grey matter volume, surface area, cortical thickness, intrinsic Gaussian curvature, mean curvature, curved index, folding index.
